# Emerging insights into the function and structure of the Integrator complex

**DOI:** 10.1080/21541264.2022.2047583

**Published:** 2022-03-20

**Authors:** Moritz M. Pfleiderer, Wojciech P. Galej

**Affiliations:** European Molecular Biology Laboratory, Grenoble, France

**Keywords:** Integrator, 3’-end processing, endonuclease, transcription attenuation, RNAPII, snRNA, cryo-EM

## Abstract

The Integrator was originally discovered as a specialized 3’-end processing endonuclease complex required for maturation of RNA polymerase II (RNAPII)-dependent small nuclear RNAs (snRNAs). Since its discovery, Integrator’s spectrum of substrates was significantly expanded to include non-polyadenylated long noncoding RNAs (lncRNA), enhancer RNAs (eRNAs), telomerase RNA (tertRNA), several Herpesvirus transcripts, and messenger RNAs (mRNAs). Recently emerging transcriptome-wide studies reveled an important role of the Integrator in protein-coding genes, where it contributes to gene expression regulation through promoter-proximal transcription attenuation. These new functional data are complemented by several structures of Integrator modules and higher-order complexes, providing mechanistic insights into Integrator-mediated processing events. In this work, we summarize recent progress in our understanding of the structure and function of the Integrator complex.

## Introduction

The Integrator complex was originally discovered as the 3’-end processing machinery required for maturation of RNA polymerase II (RNAPII)-dependent small nuclear RNAs (snRNAs)[1]. Since its discovery, additional Integrator substrates have been identified, including non-polyadenylated long noncoding RNAs (lncRNA), enhancer RNAs (eRNAs), telomerase RNA (tertRNA), several Herpesvirus transcripts, and messenger RNAs (mRNAs) [1–5]. Today, Integrator emerges as a global regulator of RNAPII activity and one of the key factors modulating expression of protein-coding genes via transcription attenuation [6–8], a mechanism tightly linked to promoter-proximal pausing of the RNAPII and the presence of the corresponding transcription factors NELF and DSIF [9–14].

Integrator consists of 14 core subunits (INTS1–INTS14) [1,15], including β-CASP/MBL endonuclease INTS11, and several other accessory factors, like the recently reported protein phosphatase complex PP2A [16–18]. Together, both enzymatic activities are specifically targeted to RNAPII via a shared Integrator scaffold, allowing for the release of the nascent transcript and “resetting” of the phosphorylation state of the RNAPII C-terminal domain by removing phosphorylation marks.

Although the composition of Integrator was well-established over the years, until recently, there was very little information about its general architecture and molecular structure [16,17]. A recent influx of crystallographic and cryo-EM data provided structural information for almost all known Integrator subunits, including the Integrator cleavage module consisting of INTS4/9/11 [18], the INTS13/14 heterodimer [19], the Integrator-PP2A complex [20], and the Integrator-PP2A bound to RNAPII [11,14]. Together, these structures provide unprecedented mechanistic insights into the spatial arrangement of Integrator’s subunits and their respective functions.

Here, we summarize the recently reported structures and discuss them in the context of our current understanding of Integrator’s function.

## Integrator acts on a diverse set of RNAPII-dependent transcripts

The Integrator complex was identified as one of the three cellular machineries performing 3’-end processing of RNAPII transcripts, alongside the **C**leavage and **P**oly**a**denylation machinery (CPA) and the **U7**-dependent **H**istone pre-mRNA **P**rocessing **M**achinery (U7-HPM) [21–23]. The CPA complex is responsible for the 3’-end cleavage and polyadenylation of the vast majority of protein-coding pre-mRNAs. An exception to that are replication-dependent histone transcripts, which are not polyadenylated, but instead processed by U7-HPM. A canonical maturation pathway for other non-polyadenylated RNAPII-dependent transcripts was unknown until Integrator was discovered [1,24].

**Figure 1.**
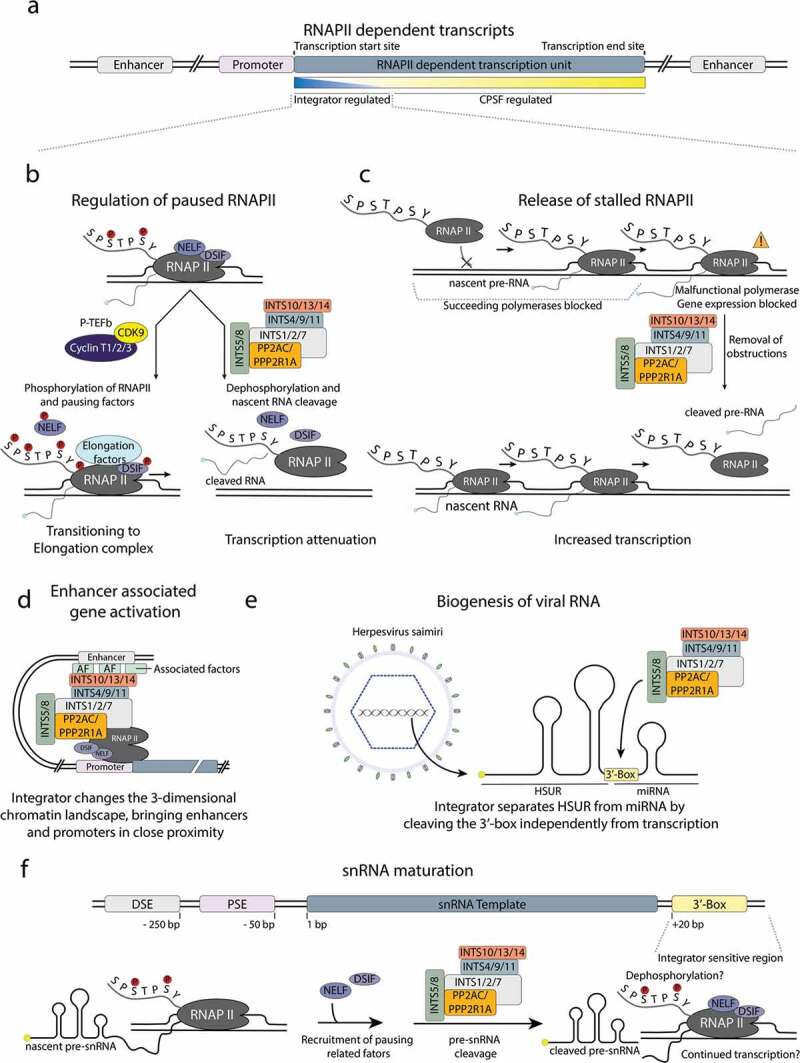
Different modes of Integrator function.

In the context of snRNAs processing, it was postulated that specific signal sequences in the pre-snRNAs would be recognized by the Integrator for further substrate processing. Indeed, mammalian U1 and U2 snRNAs share a conserved GTTTN_0-3_AAARNNAGA signal sequence (3’-box), located downstream from the mature 3’-end, which is required for their transcription termination and proper 3’-end processing [1,25,26]. Similar sequences were identified in several *Herpesvirus saimiri* Sm-class U RNA transcripts (HSURs), which were also shown to undergo Integrator-dependent processing (Figure 1) [3,5,27]. However, in this case, Integrator cleaves HSURs independently of RNAPII transcription, which stands in contrast to its activity on other RNA substrates [5]. Interestingly, in the absence of Integrator activity, cleavage and polyadenylation machinery and intrinsic properties of the sequences downstream of the snRNA coding sequences can contribute to transcription termination [28,29]. Moreover, some of the Integrator-dependent protein-coding genes contain 3’-box motifs near their transcription end sites (TESs) and were shown to have enriched RNAPII occupancy when analyzed by ChIP-seq experiments [13].

Although 3’-box sequences have been shown to be necessary for some of the Integrator-dependent 3’-end processing events, it remains unclear whether Integrator is directly involved in their recognition and whether it has any inherent sequence specificity. Biochemical studies with purified Integrator subunits could not detect any increased affinity to the substrates containing 3’-box motifs when compared to random sequences [18,19]. Recent evidence suggests that human snRNAs may exploit Integrator-sensitive gene regulation for cotranscriptional cleavage rather than recruitment to specific signal sequences [8,10].

In addition to snRNAs, Integrator was shown to be associated with several other coding and noncoding RNAPII-dependent transcription units, including human lncRNAs, tertRNA, eRNAs, and mRNAs as well as piRNAs in *C. elegans* [2,4,8,30–32]. In all cases (including snRNAs), impaired Integrator’s function results in the readthrough into neighboring transcription units and aberrant polyadenylation [8,10,30,33]. This effect is conserved among humans and different model organisms, including *C. elegans* [33], *D. melanogaster* [7], or planarian *S. mediterranea* [34].

The emerging picture suggests that all these diverse substrates could utilize a conserved mechanism of promoter-proximal transcription attenuation, which has different functional outcomes for different transcription units.

## Mechanism of integrator-mediated transcription regulation

RNAPII undergoes a highly regulated transcriptional cycle, which includes recruitment of the polymerase to the genomic locus (Initiation), transcription elongation, release of the nascent transcript via endonucleolytic cleavage (3’-end processing), and separation of the polymerase from the genomic locus (Termination).

The C-terminal domain of the largest RNAPII subunit, Rbp1 (hereafter referred to as RNAPII^CTD^), consists of multiple repeats of the heptad sequence YSPTSPS (52 repeats in humans) and is a subject to extensive phosphorylation throughout the transcription cycle [35,36]. Modification of the RNAPII^CTD^ phosphorylation state is a well-established mechanism in regulating RNAPII transcription activity, and many protein complexes, including Integrator, contain or associate with kinases and phosphatases for this purpose (e.g., transcription preinitiation complex: TFIIH; CPA: Ssu72; Mediator: CDK8).

Promoter-proximal pausing during early transcription elongation is a widely utilized regulatory mechanism in metazoans [37]. It takes place mainly within the first 40–60 nt after transcription initiation [37], when negative transcription elongation factors NELF and DSIF are recruited to the RNAPII and prevent them from further elongation [38]. Transitioning into the elongation state requires the activity of a CDK/cyclin kinase complex, P-TEFb, which phosphorylates RNAPII and pausing-related factors. This allows dissociation of NELF and recruitment of other factors to form the Elongation complex [37,39].

Mapping of the chromatin occupancy of Integrator subunits revealed a preference for promoter proximal regions, within the first 3000 bp of the transcription start site [7–9]. In line with this observation, Integrator subunits were shown to interact with the negative transcription elongation factors NELF and DSIF, which are necessary for Integrator recruitment to RNAPII on protein-coding and snRNA genes [9,10,12,13]. Coupling to RNAPII is further strengthened by recognition of specific phosphorylation marks in RNAPII^CTD^, in particular, Ser2/Ser7 [40] and Tyr1 [41].

Recruitment of Integrator to the paused RNAPII has two major consequences. First, it allows the nuclease, INTS11 to cleave and release nascent RNA. This results in small RNA fragments, which are rapidly degraded by the exosome, or in functional noncoding RNA products [6,8,11]. In addition, cleavage of nascent transcripts exposes uncapped 5’-ends of the newly synthetized RNA, which are targeted by the exonuclease Xrn2, leading to transcription termination [11,42]. Second, Integrator mediates recruitment of protein phosphatase 2 (PP2A) to the stalled polymerase complex, which allows dephosphorylation of the RNAPII^CTD^. Cross-linking data shows that Integrator binds exclusively to the C-terminal half of the RNAPII^CTD^, but it is unclear whether the entire CTD is dephosphorylated or only particular regions [11]. Integrator-associated phosphatase activity is particularly pronounced for residues Ser2, Ser5, and Ser7 [20,43]. By removing phosphorylation marks from the RNAPII^CTD^ and the associated factors (i.e., NELF and DSIF), the Integrator-PP2A complex facilitates turnover of RNAPII and transcription attenuation [44,45]. Dynamic transcription termination and reinitiation of RNAPII at gene promoters have been shown to play an important role in promoter-proximal pausing by competing with transitioning into the transcription elongation phase [46–48]. This concept is well established across different species and provides an important layer of regulation of gene expression [49–53] .

Both enzymatic functions of Integrator contribute to the same outcome, but several studies suggest that their activity is not coupled, but they work independent of each other, e.g., depletion of the nuclease module has no effect on the phosphatase function [6,20,54]. Furthermore, the phosphatase activity of Integrator seems to be dispensable for snRNA processing, as indicated by reporter assays and ChIPseq data, suggesting that snRNA processing and transcription attenuation are at least partially distinct processes [6,20,55,56].

By aborting transcription cycles, Integrator acts as a global attenuator of gene expression [7,57]. Accordingly, RNAseq experiments identify a broad range of genes that are upregulated upon Integrator depletion in *Drosophila* and in mammalian cells [6,7,57]. For a subset of genes, however, the presence of Integrator boosts transcription [6,7,57]. The first class (downregulated by Integrator) deals with genes prone to promoter-proximal pausing of RNAPII. This event takes place in only a subset of protein-coding genes, in particular, immediate early genes, even though the exact mechanisms that define pausing remain unclear [37]. Current models suggest that those genes are always transcribed but rely on external signals to progress from pausing into the elongation phase (i.e., phosphorylation by P-TEFb) and that Integrator aborts transcription at this stage (Figure 1b) [6–9,43].

The second class of genes (upregulated by Integrator) are not subjects to active Integrator-mediated downregulation, but concern the situation, when RNAPII will occasionally become stalled in a nonproductive state. These nonfunctional, stalled polymerases are also recognized by Integrator and removed, thus freeing the gene from obstructions for further transcription cycles (Figure 1c) [7,57].

By recognizing paused RNAPII, Integrator and P-TEFb are direct antagonists and compete for the same substrate of promoter proximally paused RNAPII [55]. Paused polymerase, which is recognized by P-TEFb, is phosphorylated and may transition to a productive elongation state, whereas recruitment of Integrator leads to abortive transcription (Figure 1) [55]. It is possible that the PP2-Integrator – P-TEFb – axis evolved as a phosphatase/kinase switch to fine tune transcription. Loss of both regulators results in severely decreased transcription activity as neither pause/release nor rescue of the stalled polymerase are possible [55].

In addition to its role in the promoter-proximal transcription attenuation, Integrator has been implicated in additional function near transcription end sites (TES) [13]. Under hyperosmotic stress conditions, association of the Integrator components with RNAPII is impaired, resulting in hundreds of downstream-of-gene (DoG) readthrough transcripts generated from upregulated genes [58]. Depletion of the Integrator catalytic subunit, INTS11, recapitulates this effect [58] and leads to aberrant transcription termination and 3’-end processing of a subset of protein-coding genes, even in the presence of the canonical CPA [59]. This effect is most prominent near alternative polyadenylation sites and for sequences with potential to form secondary structures [59]. Similarly, it could be shown that loss of the elongation factor SPT6 leads to readthrough in Integrator-dependent lncRNA [30]. The mechanism of the Integrator recruitment and activation at the end of genes remains to be investigated.

## Composition of the integrator complex

Integrator was originally discovered as a 12-subunit protein complex that associates with RNAPII, while searching for interactors of DSS1 (Deleted in split hand/ split foot 1), a protein that interacts directly with BRCA2 and the proteasome [1]. Two additional subunits INTS13 and INTS14 were identified in an RNAi screen performed in *D. melanogaster* [15]. Finally, Integrator was shown to recruit Protein Phosphatase 2 (PP2A), which is associated with many different protein complexes [20,60,61].

Experimental evidence suggests that the core Integrator complex can be split into multiple stable modules including the cleavage module (INTS4/9/11) [19,50], the shoulder module (INTS5/8) [19,20], and a ternary complex of INTS10/13/14 [18,45].

Several additional proteins have been associated with Integrator but are not considered part of the core complex[62]. These include the DNA damage repair factors SOSSB1/2 and SOSSC [63–65] as well as EGR1/2 and NAB2 [54]. Interestingly, the function of these associated proteins seems to differ from the canonical nuclease/phosphatase activity of the Integrator. While SOSSB1/2 and SOSSC interact with INTS3 and INTS6, they were not shown to exist in the context of the remaining twelve Integrator subunits, suggesting that the Integrator complex may consist of sometimes mutually exclusive subunits [16,66,67]. Considering that the Integrator is a highly modular protein machinery, it was postulated that it may function in multiple different compositional and conformational states and some modules may be required for additional tasks or work outside of the canonical Integrator function [15,56].

## The structure of the integrator complex

### Cleavage module – INTS4/9/11

Among all Integrator subcomplexes, the cleavage module is structurally best characterized [11,18,20,68]. While most Integrator subunits are mainly α-helical with very few predictable domain features, INTS9/INTS11 adopt MBL/β-CASP folds with remarkable similarity to their corresponding orthologs CPSF100/CPSF73 [69]. Both nucleases arrange head-to-head with a pseudo-two-fold symmetry running along their interfaces [18,20]. Their C-terminal domains (CTDs) are tightly intertwined and form an extensive interface between the two proteins, while the nuclease domains remain more loosely associated [18]. Interestingly, the CTDs of INTS9 and INTS11 can be divided into two subdomains, both of which form compact composite interfaces with one another, but only the very C-terminal CTD2 is required for dimerization [17,18]. The other composite domain CTD1 forms only upon successful dimerization of CTD2. Proper formation of this quaternary structure is required for the recruitment of INTS4, which, in turn, integrates the nuclease dimer into the fully assembled Integrator complex [18,20]. INTS4 consists of HEAT-repeats stretching along the MBL domain of INTS9 and subsequently wedging between the interface of both nucleases [18]. The C-terminal region of INTS4 forms a β-sandwich, which sits on top of the β-CASP domain of INTS9. Together, INTS4/9/11 forms a highly positively charged cavity, which serves as a conserved binding site for inositol hexaphosphate (IP6) [68]. Mutations close to the IP6-binding site cause nuclease malfunctions in reporter assays; however, it is unknown whether IP6 contributes to Integrator activity or has only a scaffolding function [18,68].

### INTS10/13/14 module

Integrator subunits 10, 13, and 14 form a stable heterotrimer, which is further associated with the cleavage module [18,19,54]. Reporter assays could show that INTS13/14 is crucial for snRNA processing in Drosophila [15]; however, in human cells, none of the three subunits appear to be critical for endonuclease activity *in vivo* [4,12,41]. Although the precise function of this module remains unknown, it was shown to play a role in activating poised enhancers during cell differentiation [54]. Studies capturing the spatial arrangement of chromatin showed that depletion of INTS13 correlates with decreased enhancer/promoter interactions [2,54]. INTS13 was also described as a factor functioning independent of the canonical Integrator and interacting with EGR1/2 and NAB2, which promote interactions with the enhancer elements [54].

Structure prediction suggests that INTS10 is a mostly α-helical protein and a crystal structure of INTS13/14 shows both proteins to form a pseudo-symmetric heterodimer with a complex interface running along all domains of both proteins [19]. Both proteins consist of a N-terminal VWA domain followed by a β-barrel, a linker, and an α-helical region, which in the case of INTS13 is further extended by a 130 residue long, flexible CTD. This region encompasses a cleavage module-binding motif (CMBM), responsible for the interaction with INTS4/9/11 [19]. The interaction of INTS10 with INTS13/14 was mapped to a MIDAS pocket in INTS14 [19]. The exact function of the INTS10/13/14 module remains elusive, but based on the structural similarity to the DNA-binding protein Ku70/80, it was proposed to be required for RNA binding [19]. However, RNA binding assays could not detect specificity or high affinity for model substrates, as observed for modules of the CPA complex [18,19,70]. To date, none of the Integrator structures provide any information about the exact position of the INTS10/13/14 module relative to the remaining subunits [11,20].

### The integrator – PP2A complex

**Figure 2.**
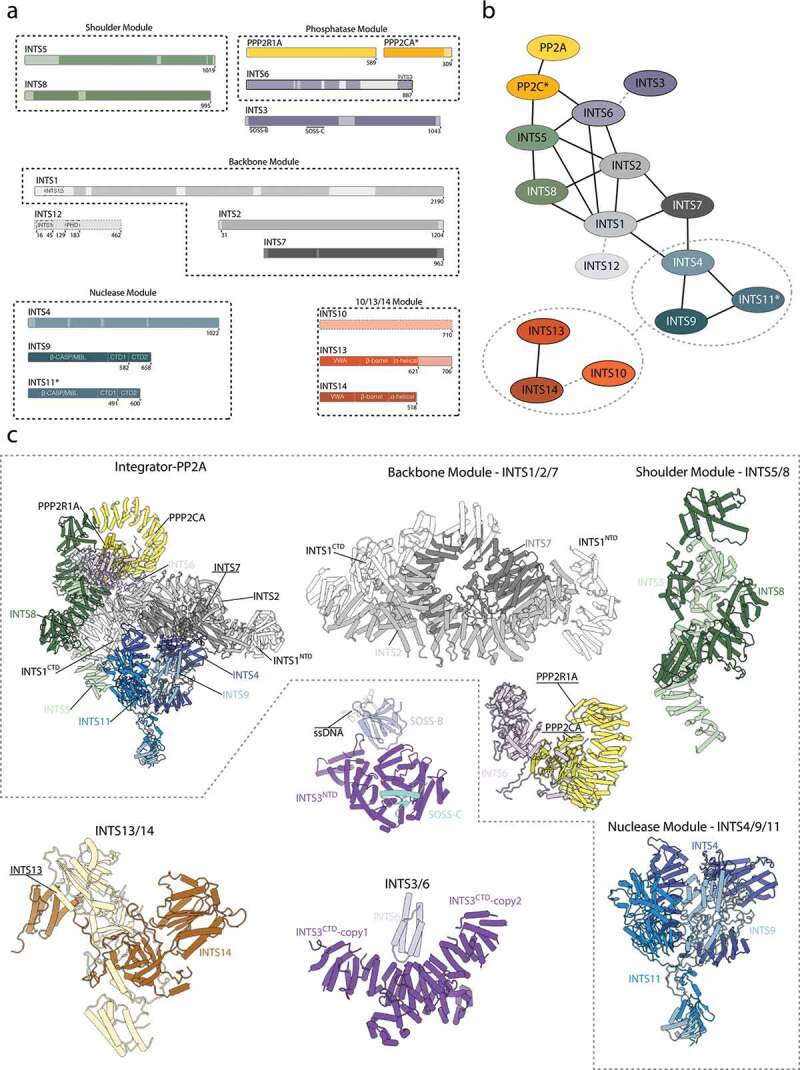
Structure of the integrator-PP2A complex.

A cryo-EM structure of the fully assembled, recombinant Integrator-PP2A complex (INTAC) revealed an overall organization of the Integrator subunits and their interactions with the accessory PP2A phosphatase complex (Figure 2) [20].

The core of the Integrator complex is composed of the subunits INTS1/2/7 forming the “backbone module”, which forms the scaffold for the nuclease (INTS4/9/11) and phosphatase modules (PPP2CA, PPP2R1A). Both enzymatic activities are located at opposing ends of the structure, separated by approximately 150 Å and seem to function independent of each other (Figure 2) [6,20].

The nuclease dimer INTS9/11 is recruited via INTS4 to INTS7 of the backbone module. INTS5/8 form the “shoulder module”, which is required for recruitment of the PP2A phosphatase to the Integrator scaffold [20,43]. PP2A family phosphatases are highly diverse protein complexes typically consisting of a catalytic subunit, a scaffolding subunit, and a regulatory subunit [61]. However, only the catalytic and scaffolding subunits are found in the Integrator [20]. Furthermore, Integrator seems to be specific for the scaffolding protein PPP2R1A and the catalytic subunit PPP2CA, whereas their homologues were detected at lower quantities [20]. PP2A is further stabilized by interactions with INTS6, which mediates contacts to INTS2 of the scaffolding module and to the shoulder module. The interaction of INTS6 with the shoulder module alone seems to be rather weak as it could not be copurified together with INTS5/8 [18]. In addition, INTS6 is a direct binding partner of INTS3 and previous interaction studies map their interface to their respective C-terminal regions [66,67,71]. Only the N-terminal region of INTS6 is ordered, and in line with this observation, INTS3 remains disordered in all available Integrator-PP2A structures [71]. INTS10/13/14 and INTS12, although present in the sample preparation, remain flexible and were not visualized in the Integrator-PP2A structure.

### Integrator – PP2A – PEC

**Figure 3.**
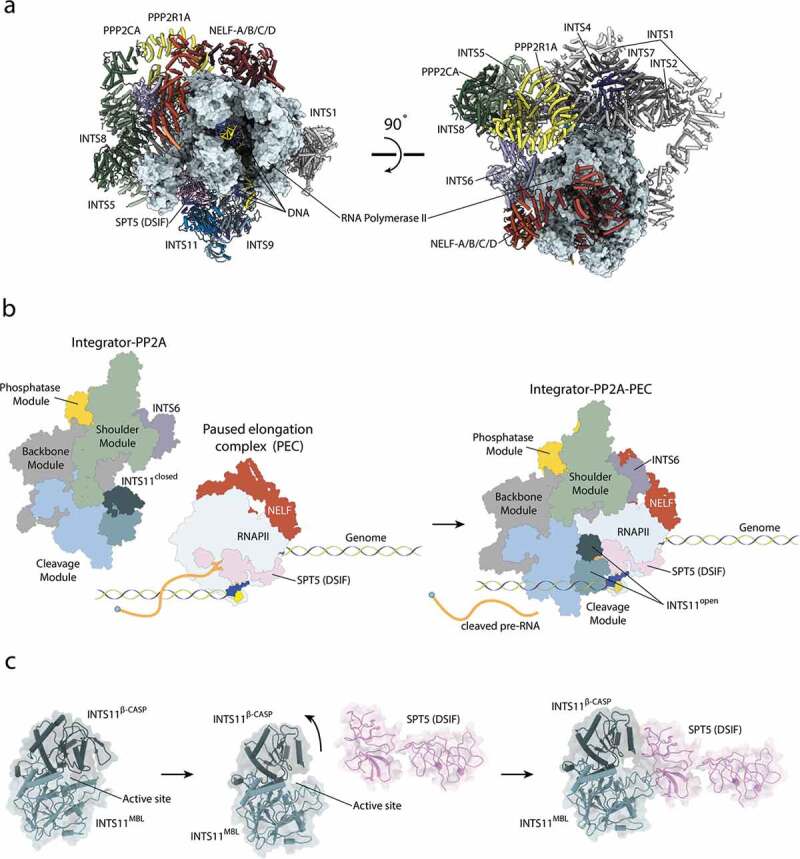
Integrator association with RNAPII and activation of the endonuclease.

Integrator is recruited specifically to RNAPII bound to NELF and DSIF (Paused Elongation Complex – PEC) [7,9,12,13,43]. The structure of the PEC alone was reported previously, and its general architecture is identical to the Integrator-bound PEC [11,38] (Figure 3). However, parts of DSIF become disordered upon the recruitment of Integrator-PP2A [11.

The Integrator wraps around the polymerase on the opposite site of the cleft with the RNAPII active site and the DNA/RNA duplex (Figure 3a). In order to accommodate RNAPII, Integrator-PP2A undergoes several subtle conformational changes, which allow it to engage with the polymerase via four interfaces [11]. The main interface is formed between the N-terminal region of INTS1 (around residues 350–600) and RBP2. The second, smaller interface depends on INTS7 binding to RBP3. Two other interactions involve transcription pausing factors, where the nuclease INTS11 binds the KOWx-4 domains of SPT5 and INTS6 to NELF-B [11].

The MBL/β-CASP domain of INTS11 is a bona fide nuclease, but remains in an inactive state until its recruitment to paused RNAPII [11,18]. Two recent cryo-EM structures [11,14] revealed that the KOWx-4 domain of SPT5 (DSIF) pushes against the β-CASP lid of INTS11 (Figure 3b-c), opening it and allowing a substrate to engage with the active center [11]. The RNA exiting the active site of RNAPII is partially protected by the KOWx-4 domain of SPT5 and is guided directly into the active site of INTS11. The distance of the active site of RNAPII to the active site of INTS11 encompasses only 20 nt [11]. Thus, the length of the released RNA depends mainly on the position of the RNAPII pause site during Integrator recruitment, in agreement with the sizes of the short, Integrator-dependent unstable transcription products [6–8,11].

Binding of RNAPII^CTD^ to the Integrator is mediated via a composite interface of INTS4/2/7, which forms a cavity that accommodates the RNAPII^CTD^ with sidechain-specific contacts. Locations of additional peptides are reported by Zheng et al. with increasing proximity to the active site of PP2A, suggesting a preferred path of the RNAPII^CTD^ within the Integrator complex [14]. However, no density could be observed close to the active site of PP2AC [11]. It is interesting to note that, while the presence of pausing factors is required for nuclease activation, RNAPII alone could be sufficient to recruit Integrator and execute its phosphatase activity.

Recruitment of Integrator is mutually exclusive with the recruitment of the Mediator complex and other factors forming the transcription preinitiation complex (PIC). Similarly, other transcription factors like PAF1C or SPT6 are not compatible with the presence of the Integrator [11,14,72].

### INTS3 and DNA damage repair complexes

INTS3 is a well-established subunit of a DNA damage repair complex, SOSS (sensor of ssDNA), that acts autonomously from Integrator. N-terminal α-helical repeats of INTS3 form the scaffold for the two interactors SOSSB1/2 (alternatively hSSB1 – human ssDNA binding protein 1/2) and C9ORF80 (SOSS-C, MISE – minute INTS3/hSSB1-associated element, INIP – INTS3 interacting protein) [63–65]. SOSS was described before as a major factor in maintaining genome stability, aiding detection of DNA lesions and recruiting factors downstream of the DNA damage repair pathway [63,71,73]. INTS3 interaction with SOSS-B and SOSS-C is sensitive to UV-light exposure, which as well functions as a signal for transport into the nucleus and localization to chromatin [74]. A crystal structure of the INTS3^NTD^ together with SOSS-B, SOSS-C, and ssDNA could show that SOSS-B1 adopts an oligonucleotide binding fold, interacting with the bases of a 10 nt long ssDNA [16]. The function of SOSS-C remains elusive as it is dispensable for DNA binding [16]. Biochemical studies and a crystal structure of the INTS3^CTD^ identified an additional, putative nucleic acid binding site with a μM affinity and preference for RNA [66,75].

In addition, a direct interaction of INTS3 and INTS6 was reported [63]. Immunoprecipitation experiments and a crystal structure could show that the CTD of INTS3 mediates interaction with INTS6 and may be involved in multimerization [66,67]. Based on these interaction studies, it is likely that the heterotrimeric complex of INTS3/INTS6/hSSB1 functions independent of the Integrator [63]. However, it cannot be excluded that this module is only weakly tethered to the fully assembled Integrator and is only required under certain conditions.

## Common design principles of the 3’-end processing machineries

3’-end processing is a crucial step in gene expression, and factors involved in it are often highly conserved and fine-tuned to recognize their cognate substrates. Among them, the CPA complex is conserved from yeast to humans [22], while the U7-HPM and Integrator are restricted to metazoans [76].

Nonetheless, several features are common to all three 3’-end processing machineries like the catalytic core composed of an active and inactive MBL/β-CASP nucleases [18,77]. CPA and U7-HPM share the same catalytic core composed of CPSF73 and its inactive partner CPSF100 [22], while Integrator uses the homologous INTS11 and inactive INTS9, respectively. Integrator does not share any subunits with the other two machineries, nor does it have any homologous subunits, besides the INTS9/11 nuclease heterodimer [1]. Both, CPSF100/73 and INTS9/11, nuclease heterodimers are arranged in a head-to-head orientation with a pseudo two-fold symmetry axis running along their dimerization interface. Stable interaction is mainly driven by their respective CTDs, which are tightly intertwined and form two composite domains, CTD1 and CTD2.

Noteworthily, the inactive nucleases, CPSF100 or INTS9, contain noncanonical insertions in their respective pseudonuclease domains. In the case of INTS9, the NAD (Nine Accessory Domain) is composed of two insertions in the MBL domain, which forms a compact domain [18]. In contrast, the CPSF100 insertion is mostly unstructured and is located in the β-CASP lid [78]. However, it contains a short signal peptide (PIM – Polyadenylation Specificity Factor Interacting Motif), which tethers it to the polyadenylation specificity factor, the protein complex that recognizes polyadenylation signal sequences in the nascent pre-mRNA [78].

The nuclease catalytic subunits, INTS11 and CPSF73, are kept in inactivated states and require additional factors to achieve catalytic competence. As described earlier, Integrator undergoes activation through association with promoter-proximally paused RNAPII, where negative transcription elongation factor SPT5 pushes into the β-CASP lid of INTS11, displacing it and making the active center accessible [11,14]. U7-HPM utilizes a different mechanism, in which a protein from the LSm ring (i.e., Lsm10) inserts between the MBL and β-CASP domain of CPSF73 and allows the RNA substrate to engage with the active site [78]. The fact that the LSm ring associates with the U7 snRNA, which is responsible for histone pre-mRNA recognition, links the catalytic activation with the substrate recognition. However, Lsm10 is not present in the CPA, implying that it must utilize a different activation mechanism. Recent studies show that Rbbp6 or its yeast homolog Mpe1 is required for CPA catalytic activation [79–82]. To date, no structural information is available for the activated nuclease CPSF73 in the context of the CPA, and thus, the exact mechanism of the active site opening mechanism remains elusive.

It is interesting to note that both CPA and Integrator incorporate nuclease and phosphatase activities, which is not the case for the U7-HPM. This is consistent with the fact that Integrator and CPA interact directly with the RNAPII^CTD^ to regulate its phosphorylation states, while a similar interaction seems to be missing in U7-HPM-mediated 3´-end processing. In the CPA, Symplekin recruits the phosphatase Ssu72 to the complex. Although Symplekin is shared between CPA and U7-HPM, the Ssu72 binding interface is unavailable in the U7-HPM, which exemplifies the specialization of both machineries [77,83].

The highly conserved architecture of the cleavage modules suggests a common evolutionary origin of the core of all 3’-end processing machineries. Additional subunits, which are not shared between different machineries, were likely recruited to each complex during adaptation to specific tasks.

Functionally, the main difference in Integrator and other 3’-end processing factors is that Integrator nuclease and phosphatase activity has a mainly abortive impact on transcription, while CPA and the U7-HPM are key factors required for the maturation of transcripts. Exceptions like snRNA exploit Integrator-sensitive regulation to achieve different functional outcomes [8].

## Integrator involvement in development and cell differentiation

Multiple studies show that impaired Integrator function disrupts the fine-tuned equilibrium of specific transcripts required for cell differentiation, resulting in disease relevant phenotypes on a cellular level and in whole organisms. These effects are conserved among different species [84].

In *Drosophila*, Integrator has been implicated in the differentiation process of neuronal progenitor cells [85]. The study identified more than 1400 target genes regulated by INTS5, including transcription factors such as CycE and Notch signaling components, previously proposed to be involved in cell differentiation [85].

Similar results were reproduced in Zebrafish, where Integrator was shown to be crucial for embryonic development [86]. Here, INTS6 was shown to be a central factor in regulating dorsoventral patterning by disrupting expression of crucial signaling factors such as BMP ligands and mediators of the Wnt signaling pathway [86].

In mouse models, Integrator was shown to regulate the differentiation of adipocytes [87]. Depletion of Integrator subunits resulted in altered mRNA levels. In addition, the authors were able to correlate INTS expression with the process of differentiation pointing at the Integrator as a key factor in its regulation. Another study was able to show that Integrator is required for hematopoiesis [88]. Homozygous deletion of most INTS in mice is lethal in preweaning states, further highlighting the importance of Integrator in development [89].

## Integrator malfunctions and genetic disorders in humans

The Integrator complex is involved in regulation of transcription homeostasis in human cells, in particular, in development and under stress conditions, and therefore, it is not surprising that its malfunctions are associated with genetic disorders and cancer [6,55,84].

In 2017, first studies emerged linking neurodegenerative disorders to mutations in INTS1 and INTS8 in several individuals from different families [90]. In all cases, biallelic Integrator mutations resulted in severe phenotypes with strongly impaired cognitive abilities [90–92]. For INTS8, one allele produced an unstable RNA due to a missense mutation, whereas the second allele contained a 3-residue deletion, which was shown to be the predominant protein in the patients. Comparing Integrator assembly in the mutated or WT variant of INTS8 showed a significant reduction in the enrichment of other INTS in human tissue culture [90]. Consistent with biochemical data, patients lacking functional INTS8 showed changes in splicing patterns and several transcripts were significantly up- or downregulated compared to healthy individuals [7,8,90]. Similarly, the mutation of INTS1 could be associated with a strong reduction of mRNA levels in skin fibroblasts [90].

A detailed study evaluating the genome and transcriptome of different cancer types revealed that all 14 Integrator subunits are frequently mutated in cancer cells [93] and disregulate various cellular functions [94,95]. However, as several INTS are part of the genome maintenance machinery, possibly outside of the canonical Integrator function, it is difficult to establish clear causative links between those observations [96–98].

By regulating a large subset of RNAPII-dependent transcripts, Integrator is in principle capable of affecting various aspects of cell function, explaining its involvement in sometimes seemingly unrelated processes.

## Open questions and future perspectives

Since Integrator was discovered, our understanding of its cellular function has undergone a paradigm shift. It was originally thought to be a specialized 3’-end processing machinery required for a very specific set of substrates, but recently emerged as a global regulator of the RNAPII activity [1,8]. Functional data are backed up by a gallery of different structures providing detailed insights into the exact spatial arrangement of the different subunits, their recruitment to RNAs, and activation of the nuclease subunit [11,18–20]. Nonetheless, several aspects of Integrator function remain elusive.

First, it is still unknown how Integrator recruitment to paused RNAPII is regulated. In general, P-TEFb and Integrator are present together in cells and compete for access to the stalled RNAPII. The modulation of this competition, in particular upon activation of signaling pathways and across different cell types, remains to be investigated.

Second, our current structural understanding of Integrator activity allows for assignment of functions to almost all Integrator subunits with exception of INTS3, INTS12, and INTS10/13/14.

INTS3 is also part of a DNA damage repair complex, but its functions here or in transcription attenuation remain elusive. It is unclear how the INTS3/6/SOSSB/SOSSC module relates to the Integrator complex and what is the function of INTS3 without its binding partners SOSSB and SOSSC.

INTS12 is a largely unstructured protein, which supports the stability of INTS1 and may be required for recruitment of RNAPII [11,99]. Furthermore, the exact function of INTS10/13/14 remains undetermined. It was proposed to be involved in DNA/RNA binding and to be required for enhancer binding together with other accessory factors [54]. However, it is not clear how the function of this module corresponds to the activity of the phosphatase and the nuclease modules and whether this module is required for other Integrator functions.

Finally, Integrator’s activity in the context of Herpes viruses seems to be largely independent of the transcription machinery. This modus operandi stands in contrast to our current understanding of Integrator activity. It remains unclear how Integrator achieves substrate specificity and nuclease activation in the absence of RNAPII and its pausing factor SPT5 (DSIF). In either case, it is possible that yet undiscovered factors are necessary to regulate Integrator activity under specific circumstances. Identifying different variants of Integrator modules and their impact on different aspects of the Integrator function will be one the main goals of future research directions.

**a)** Schematic representation of an RNAPII-dependent transcriptional unit with the promoter proximal regions sensitive to Integrator regulation. **b)** Possible fates of paused RNAPII. Recruitment of the Integrator results in cleavage of the nascent RNA and dephosphorylation of the RNAPII, leading to transcription attenuation. Recruitment of P-TEFb promotes transitioning into a transcription elongation complex. **c)** Integrator-mediated removal of stalled RNAPII frees the target gene from obstructions and facilitates further cycles of transcription. **d)** Integrator-mediated transcription regulation by affecting the 3-dimensional chromatin landscape, bringing enhancer and promoter elements into close proximity to induce transcription. **e)** Processing of *Herpesvirus saimiri* RNAs by the host Integrator is independent of RNAPII. **f)** Cartoon representation of an snRNA gene with associated regulatory elements and the indicated region sensitive to Integrator processing. Pausing-related factors are recruited and facilitate Integrator-mediated release of the pre-snRNA.

**a)** Sequence bar representation of all subunits forming the Integrator-PP2A complex. Regions with structural information available are opaque, transparent regions, and dotted lines correspond to sequence regions, which are not resolved in the reported cryo-EM/X-ray experiments. No structural information is available for INTS10 and INTS12. **b)** Diagram showing the interaction network of the different Integrator subunits. Dark lines correspond to interfaces confirmed by structural data, and dotted lines are contacts inferred from biochemical data. **c)** Structure gallery showing all known Integrator structures. Figures based on 7CUN (Integrator-PPA complex) [20], 6SN1 (INTS13/14) [19], 7BV7 (INTS3/6) [67], and 4OWW (INTS3/SOSS-A/SOSS-B) [16].

**a)** Recruitment of Integrator to RNAPII. RNAPII is shown in surface representation. Integrator and pausing factors NELF and DSIF are depicted as colored cartoons [11]. **b)** Mechanism of the Integrator recruitment to paused RNAPII and activation of the INTS11 endonuclease. **c)** A Close-up view of INTS11 and its interaction with SPT5 shown in the same orientation as in b). Figures are based on PDB coordinates: 7BFP [18] and 7PKS [11].
